# Identification and validation of signal recognition particle 14 as a prognostic biomarker predicting overall survival in patients with acute myeloid leukemia

**DOI:** 10.1186/s12920-021-00975-2

**Published:** 2021-05-13

**Authors:** Lingling Shi, Rui Huang, Yongrong Lai

**Affiliations:** Department of Hematology, The First Affiliated Hospital of Guangxi Medical University, Shuang Yong Road 6, Nanning, 530021 Guangxi People’s Republic of China

**Keywords:** Signal recognition particle 14, Acute myeloid leukemia, Prognosis, GSE12417, The Cancer Genome Atlas

## Abstract

**Background:**

This study aimed to determine and verify the prognostic value and potential functional mechanism of signal recognition particle 14 (SRP14) in acute myeloid leukemia (AML) using a genome-wide expression profile dataset.

**Methods:**

We obtained an AML genome-wide expression profile dataset and clinical prognostic data from The Cancer Genome Atlas (TCGA) and GSE12417 databases, and explored the prognostic value and functional mechanism of SRP14 in AML using survival analysis and various online tools.

**Results:**

Survival analysis showed that AML patients with high SRP14 expression had poorer overall survival than patients with low SRP14 expression. Time-dependent receiver operating characteristic curves indicated that SRP14 had good accuracy for predicting the prognosis in patients with AML. Genome-wide co-expression analysis suggested that SRP14 may play a role in AML by participating in the regulation of biological processes and signaling pathways, such as cell cycle, cell adhesion, mitogen-activated protein kinase, tumor necrosis factor, T cell receptor, DNA damage response, and nuclear factor-kappa B (NF-κB) signaling. Gene set enrichment analysis indicated that SRP14 was significantly enriched in biological processes and signaling pathways including regulation of hematopoietic progenitor cell differentiation and stem cell differentiation, intrinsic apoptotic signaling pathway by p53 class mediator, interleukin-1, T cell mediated cytotoxicity, and NF-κB-inducing kinase/NF-κB signaling. Using the TCGA AML dataset, we also identified four drugs (phenazone, benzydamine, cinnarizine, antazoline) that may serve as SRP14-targeted drugs in AML.

**Conclusion:**

The current results revealed that high SRP14 expression was significantly related to a poor prognosis and may serve as a prognostic biomarker in patients with AML.

**Supplementary Information:**

The online version contains supplementary material available at 10.1186/s12920-021-00975-2.

## Background

Acute myeloid leukemia (AML) comprises a heterogeneous group of diseases characterized by the uncontrolled proliferation of myeloid precursor cells, which gradually replaces normal bone marrow hematopoiesis [1]. The genetic changes in the tumor clones lead to a cascade of molecular events, which in turn cause abnormal proliferation and differentiation of malignant cells and inhibit normal hematopoiesis. The identification of cytogenetic changes plays an increasingly important role in the diagnosis of AML, and in its prognosis prediction and treatment strategy formulation [2]. Gene expression profiling has been widely used in AML, and the resulting gene profiles can assist in the typing diagnosis and in assessing the prognostic risk and chemotherapeutic drug resistance. These applications require high-throughput sequencing to screen and identify AML-related biomarkers. The most effective approach involves preliminary screening and identification of high-throughput sequencing datasets based on the Gene Expression Omnibus (GEO) and The Cancer Genome Atlas (TCGA) databases. Using GEO database retrieval, we found that the GSE12417 cohort dataset contained both an AML bone marrow expression profile dataset and prognostic data. We therefore used the GSE12417 and TCGA AML cohorts for further investigation. The expression of signal recognition particle 14 (SRP14) has previously been reported to be closely related to endometrial cancer [3] and to serve as a reference gene for AML when combined with other genes [4]. However, the prognostic value of SRP14 in AML has not been reported. The main purpose of this study was to identify and verify the prognostic value and potential functional mechanism of SRP14 in AML using a genome-wide expression profile dataset.

## Methods

### Dataset collection and processing

The genome-wide expression profile chip datasets were derived from the TCGA LAML (https://portal.gdc.cancer.gov) and GSE12417 Affymetrix Human Genome U133A Array cohorts (https://www.ncbi.nlm.nih.gov/geo/query/acc.cgi?acc=GSE12417), respectively [5, 6]. AML patients from TCGA served as a training cohort, while GSE12417 patients served as a validation cohort. TCGA cohort RNA-seq dataset were normalized using edgeR and GSE12417 expression profile chip data were normalized using limma [7, 8]. In the case of multiple probes corresponding to one gene in the GSE12417 cohort, the average value of the probes was taken to represent the expression level of this gene. After excluding patients without prognostic information or RNA sequencing data, we included a total of 130 patients with AML in the survival analysis, and 160 AML patients from the GSE12417 cohort were used for follow-up survival analysis. All the data in this study were sourced from open access databases. No experiments involving animals or humans were conducted and additional ethics committee approval was therefore not required.

### Survival analysis of SRP14 in AML

High and low SRP14 expression groups were defined by a cut-off based on the median level of SRP14 expression in the cohort. We compared the prognosis of AML patients in relation to SRP14 expression levels using Kaplan–Meier analysis. We also evaluated the accuracy of SRP14 for AML prognosis prediction using the SurvivalROC (https://cran.r-project.org/web/packages/survivalROC/index.html) package.

### Functional mechanism of SRP14 in AML

We explored the functional mechanism of SRP14 in AML by screening SRP14 co-expressed genes for functional enrichment and by gene set enrichment analysis (GSEA; http://software.broadinstitute.org/gsea/index.jsp) approaches. Genes co-expressed with SRP14 in AML were identified using the cor function and whole genome expression profile datasets in the R platform. Gene Ontology (GO) terms and Kyoto Encyclopedia of Genes and Genomes enrichment were analyzed using the Database for Annotation, Visualization, and Integrated Discovery v6.8 (DAVID v6.8, https://david.ncifcrf.gov/home.jsp) tool [9]. The visualized interaction networks of SRP14 and its co-expressed genes were generated using Cytoscape version 3.6.1 software. GSEA was also used for SRP14 functional mechanism mining. The C2 (c2.all.v7.0.symbols.gmt) and C5 (c5.all.v7.0.symbols.gmt) gene sets, from the Explore the Molecular Signatures Database (MSigDB, https://www.gsea-msigdb.org/gsea/msigdb/index.jsp), were used as reference sets for GSEA analysis [10]. We considered |normalized enrichment score (NES)|> 1, nominal *P* < 0.05, and a false discovery rate (FDR) < 0.25 to be statistically significant.

### Targeted drug screening of SRP14 in AML

We screened for SRP14-targeted drugs in AML using the Connectivity Map (CMap) online tool for targeted drug screening [11]. We defined differentially expressed genes (DEGs) according to the criteria |log2 fold change (FC)|> 2, *P* < 0.05, and FDR < 0.05. DEG screening of the TCGA and GSE12417 cohorts were carried out using the edgeR and limma packages in the R platform, respectively. The chemical structures of the targeting drugs were downloaded from PubChem (https://pubchem.ncbi.nlm.nih.gov), and drug–gene interaction networks were generated using STITCH (http://stitch.embl.de/) [12, 13].

### Statistical analysis

Multiple testing in GSEA analysis and DEG screening were corrected according to the FDR method. Kaplan–Meier survival curves were compared using log rank tests. Hazard ratios (HRs) and 95% confidence interval (CIs) were used to compare the risk ratios of survival differences between different subgroups. The ggplot2 (https://cran.r-project.org/web/packages/ggplot2/index.html) package was used for visualization mapping. Analyses were carried out using R platform version 3.6.2. *P* < 0.05 was considered to be statistically significant.

## Results

### Survival analysis of SRP14 in AML

**Fig. 1:**
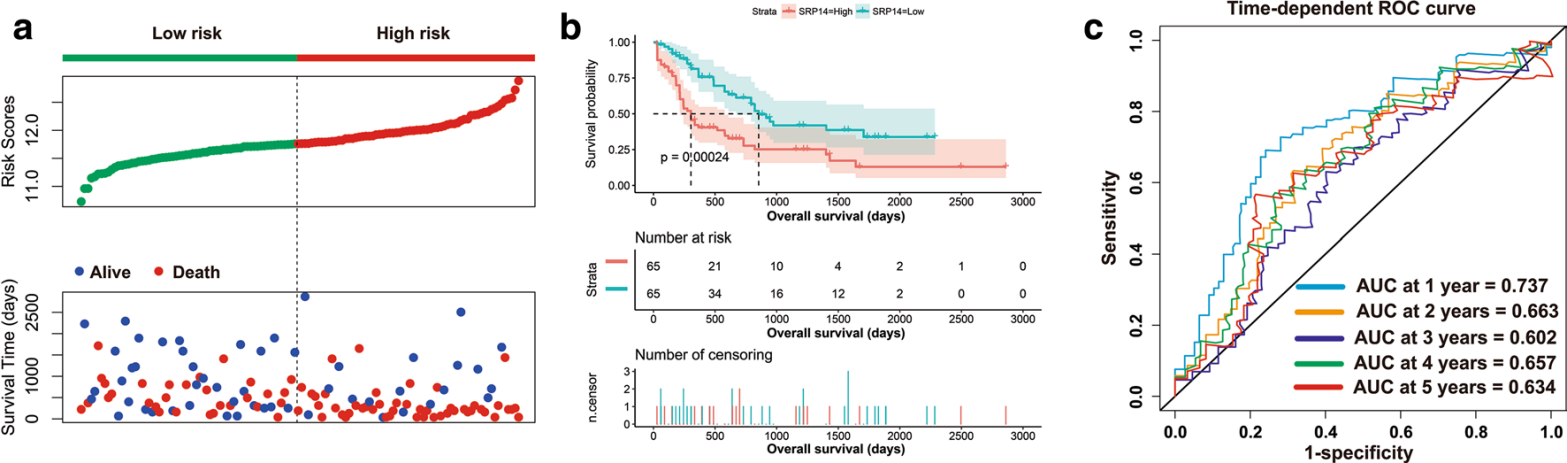
Prognostic analysis of SRP14 in the TCGA cohort. **a** Scatterplot of SRP14 expression and patient survival time distribution; **b** Kaplan–Meier survival curve of SRP14; **c** time-dependent ROC curve of SRP14

**Fig. 2:**
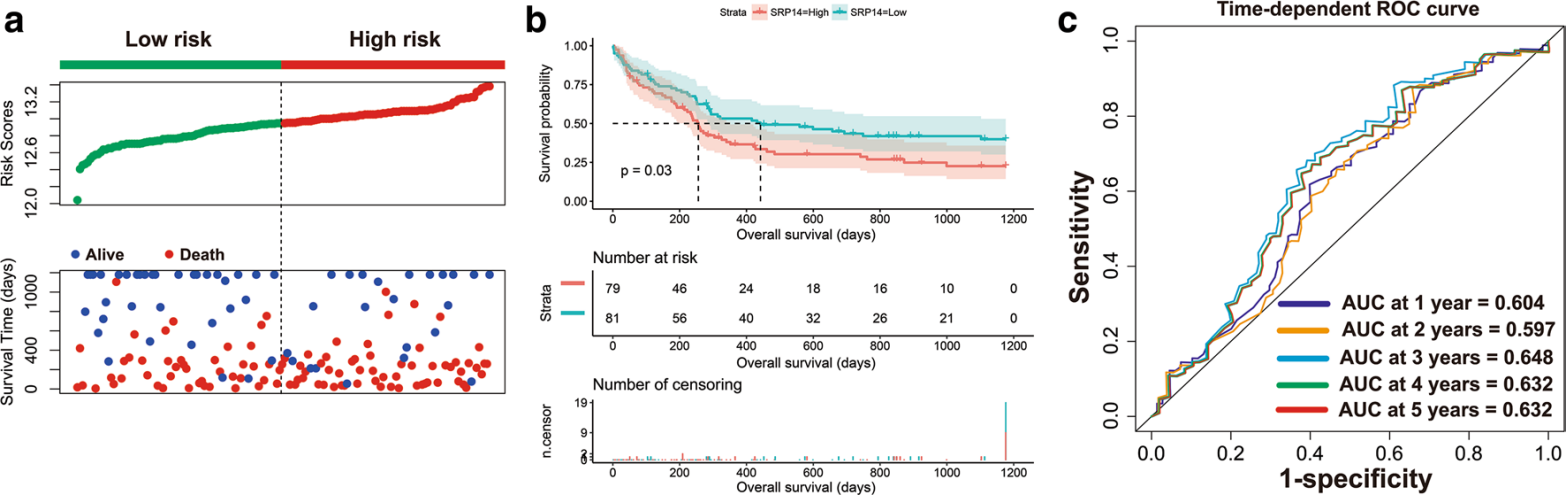
Prognostic analysis of SRP14 in the GSE12417 cohort. **a** Scatterplot of SRP14 expression and patient survival time distribution; **b** Kaplan–Meier survival curve of SRP14; **c** time-dependent ROC curve of SRP14

Survival analysis showed that AML patients with high SRP14 expression had shorter overall survival (OS) than patients with low SRP14 expression (log-rank *P* = 0.00024) (Fig. 1a, b). The median survival time (MST) in the high-SRP14 expression group was 304 days and the MST in the low-SRP14 expression group was 854 days. High SRP14 expression was associated with a significantly higher risk of death (HR = 2.288, 95%CI = 1.448–3.614). The time-dependent receiver operating characteristic (ROC) survival curve indicated that SRP14 had the highest accuracy for prognostic prediction in the TCGA AML cohort at 1 year, with an area under the curve (AUC) of 0.737 (Fig. 1c), while its accuracy for predicting 5-year survival was 0.634 (Fig. 1c). Similar results were observed in the GSE12417 cohort, AML patients with high SRP14 expression had a shorter OS than patients with low SRP14 expression (log-rank *P* = 0.03) (Fig. 2a, b). The MST in the high-SRP14 expression group was 256 days, compared with 442 days in the low-SRP14 group. AML patients with high SRP14 expression had a higher risk of death (HR = 1.536, 95%CI = 1.038–2.273). The time-dependent ROC survival curve indicated that SRP14 had the highest accuracy for prognostic prediction in AML patients in the GSE12417 cohort at 3 years, with an AUC of 0.648 (Fig. 2c), while its accuracy for predicting 5-year survival was 0.632 (Fig. 2c).

### Screening and functional enrichment of SRP14 co-expressed genes

**Fig. 3:**
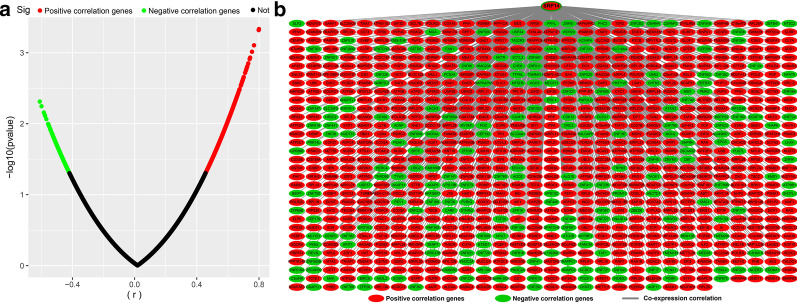
Interaction network and distribution map of SRP14 co-expressed genes in the TCGA cohort. **a** Volcano plot of SRP14 co-expressed genes; **b** interaction network of SRP14 and its co-expressed genes

**Fig. 4:**
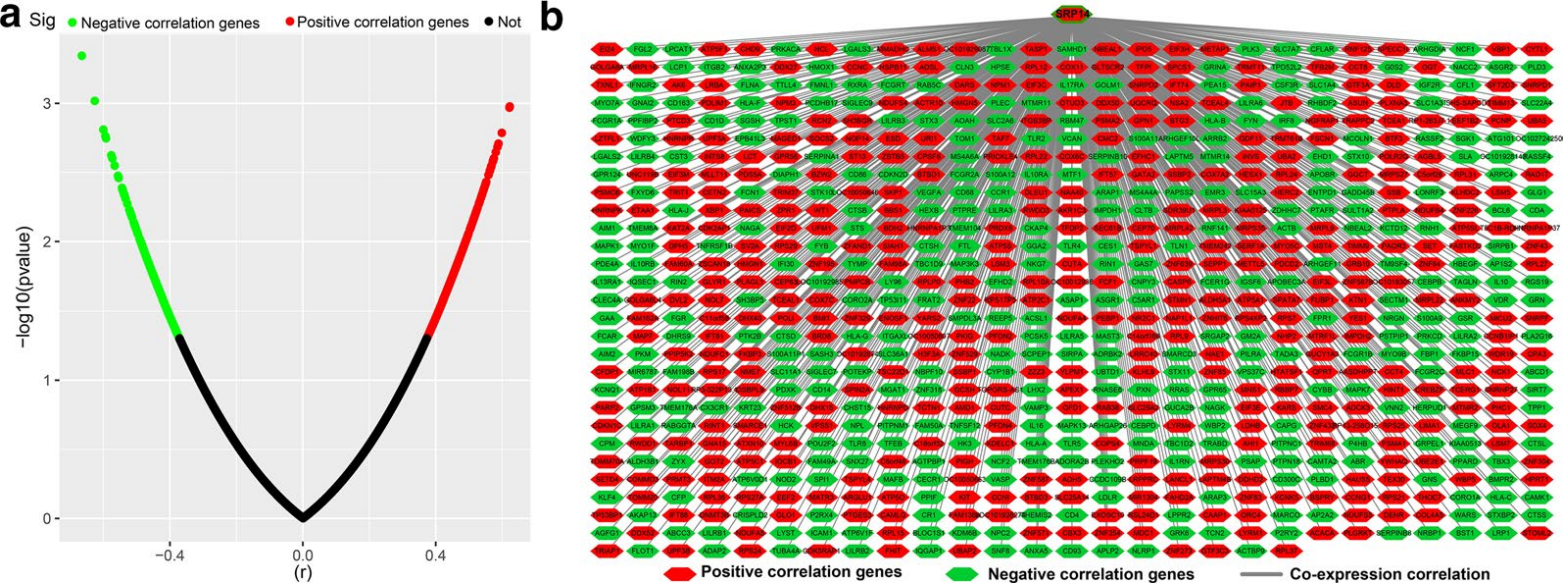
Interaction network and distribution map of SRP14 co-expressed genes in the GSE12417 cohort. **a** Volcano plot of SRP14 co-expressed genes; **b** interaction network of SRP14 and its co-expressed genes

**Fig. 5:**
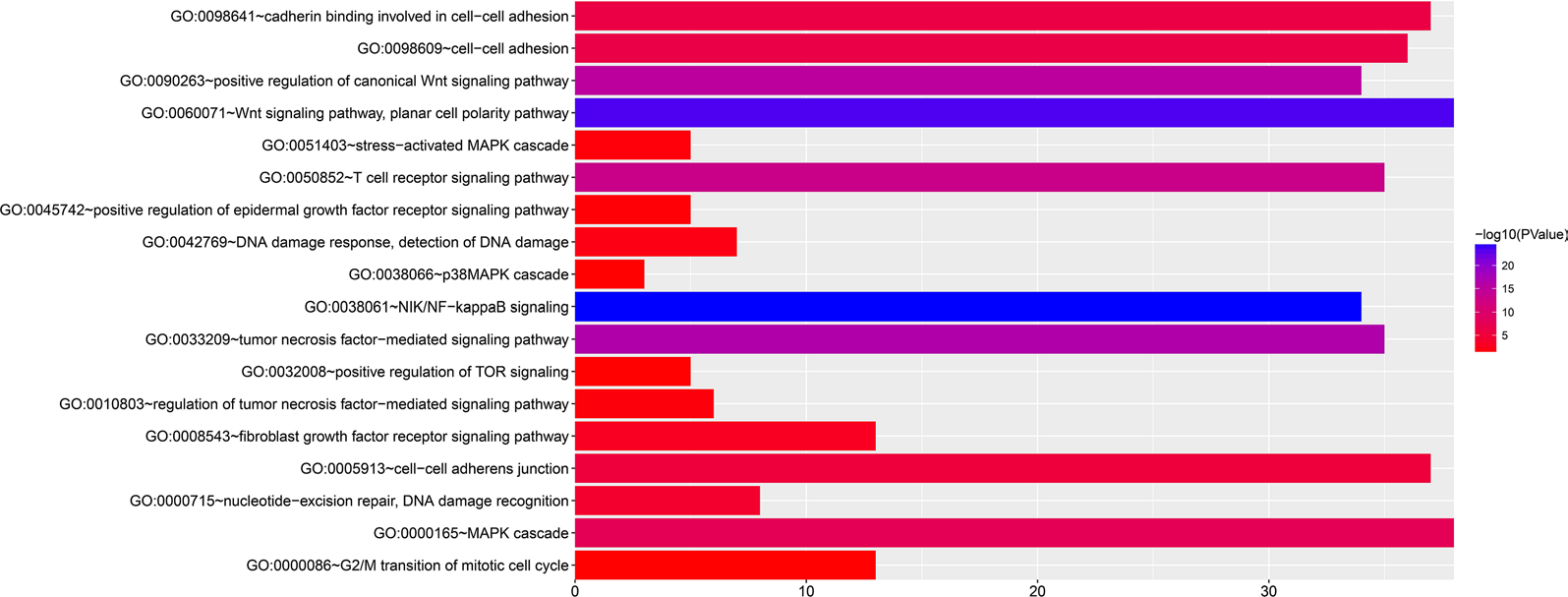
Functional enrichment analysis results of SRP14 and its co-expressed genes in TCGA cohort

**Fig. 6:**
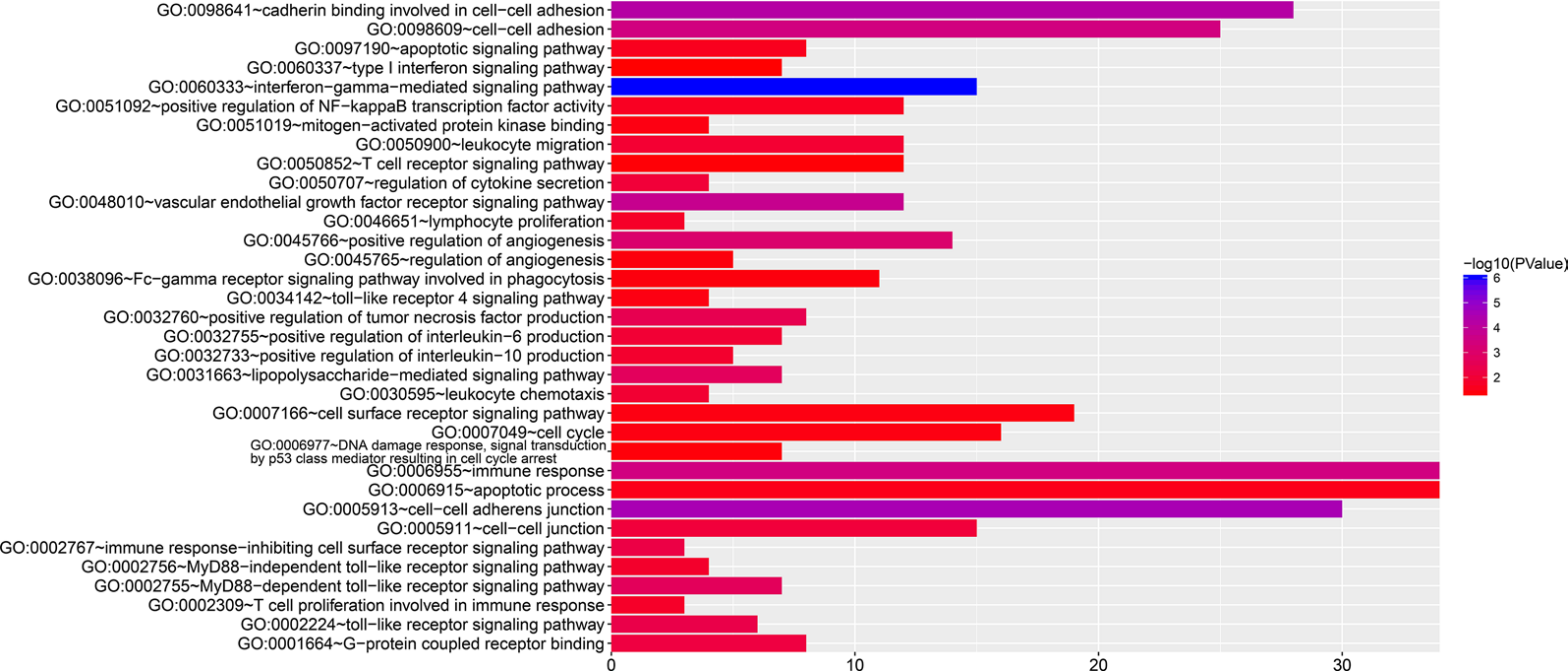
Functional enrichment analysis results of SRP14 and its co-expressed genes in GSE12417 cohort

**Fig. 7:**
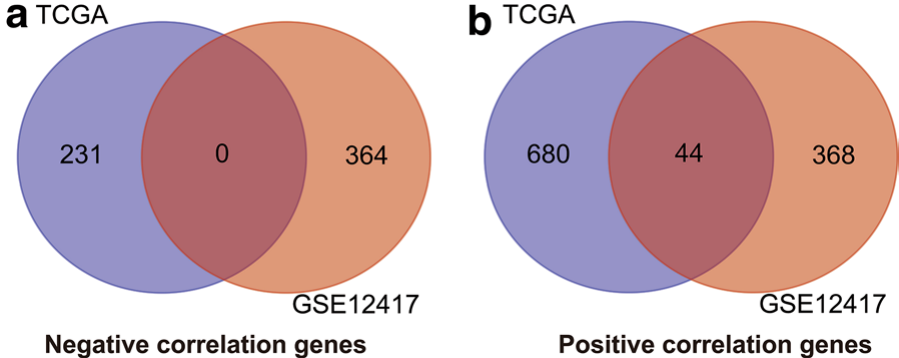
Venn plot of the intersection of SRP14 co-expressed genes between the TCGA and GSE12417 cohorts. **a** Venn plot of the intersection of SRP14 negative co-expressed genes; **b** Venn plot of the intersection of SRP14 positive co-expressed genes

Using the genome-wide expression profiling dataset, we screened 955 SRP14 co-expressed genes in the TCGA AML cohort, including 724 positively correlated and 231 negatively correlated genes (Fig. 3a, b, Additional file 2: Table S1). We also screened a total of 776 SRP14 co-expressed genes in the GSE12417 AML cohort, including 412 positively correlated and 364 negatively correlated genes (Fig. 4a, b, Additional file 2: Table S2). Functional enrichment analysis suggested that SRP14 and its co-expressed genes screened in the TCGA AML cohort could be significantly involved in biological processes including mitogen-activate protein kinase (MAPK) cascade, cell–cell adhesion, cadherin binding involved in cell–cell adhesion, DNA damage response, detection of DNA damage, stress-activated MAPK cascade, p38/MAPK cascade, G2/M transition of mitotic cell cycle and nucleotide-excision repair, and DNA damage recognition (Fig. 5, Additional file 2: Table S3), as well as in signaling pathways including NF-κB-inducing kinase (NIK)/nuclear factor-κB (NF-κB) signaling, Wnt, T cell receptor (TCR), fibroblast growth factor receptor (FGFR), epidermal growth factor receptor, and tumor necrosis factor (TNF)-mediated signaling (Fig. 5, Additional file 2: Table S3). Functional enrichment analysis suggested that SRP14 and its co-expressed genes screened in the GSE12417 AML cohort could be significantly involved in biological processes including cell–cell adhesion, immune response, cadherin binding involved in cell–cell adhesion, regulation of angiogenesis, G-protein coupled receptor binding, regulation of cytokine secretion, positive regulation of interleukin (IL)-6 production, leukocyte migration, apoptotic process, MAPK binding, cell cycle, positive regulation of NF-κB transcription factor activity and DNA damage response, and signal transduction by p53 class mediator resulting in cell cycle arrest (Fig. 6, Additional file 2: Table S4), and in signaling pathways including vascular endothelial growth factor receptor, MyD88-dependent toll-like receptor, Toll-like receptor (TLR), immune response-inhibiting cell surface receptor, MyD88-independent TLR, apoptotic, cell surface receptor, TLR4, TCR, type I interferon, and lipopolysaccharide-mediated and interferon-gamma-mediated signaling (Fig. 6, Additional file 2: Table S4). We identified 44 SRP14 co-expressed genes selected by both cohorts (Fig. 7). Functional enrichment analysis suggested that these 44 genes were significantly involved in SRP-dependent co-translational protein targeting to the membrane, mitochondrial proton-transporting ATP synthase complex, ATP synthesis-coupled proton transport, cytochrome-c oxidase activity, extracellular matrix, ATPase activity, mRNA splicing, via spliceosome, focal adhesion, mitochondrial respiratory chain complex IV, mitochondrial electron transport, cytochrome c to oxygen, oxidative phosphorylation, catalytic step 2 spliceosome, nuclear-transcribed mRNA catabolic process, nonsense-mediated decay, methylosome, and erythrocyte homeostasis (Additional file 2: Table S5).

### GSEA

**Fig. 8:**
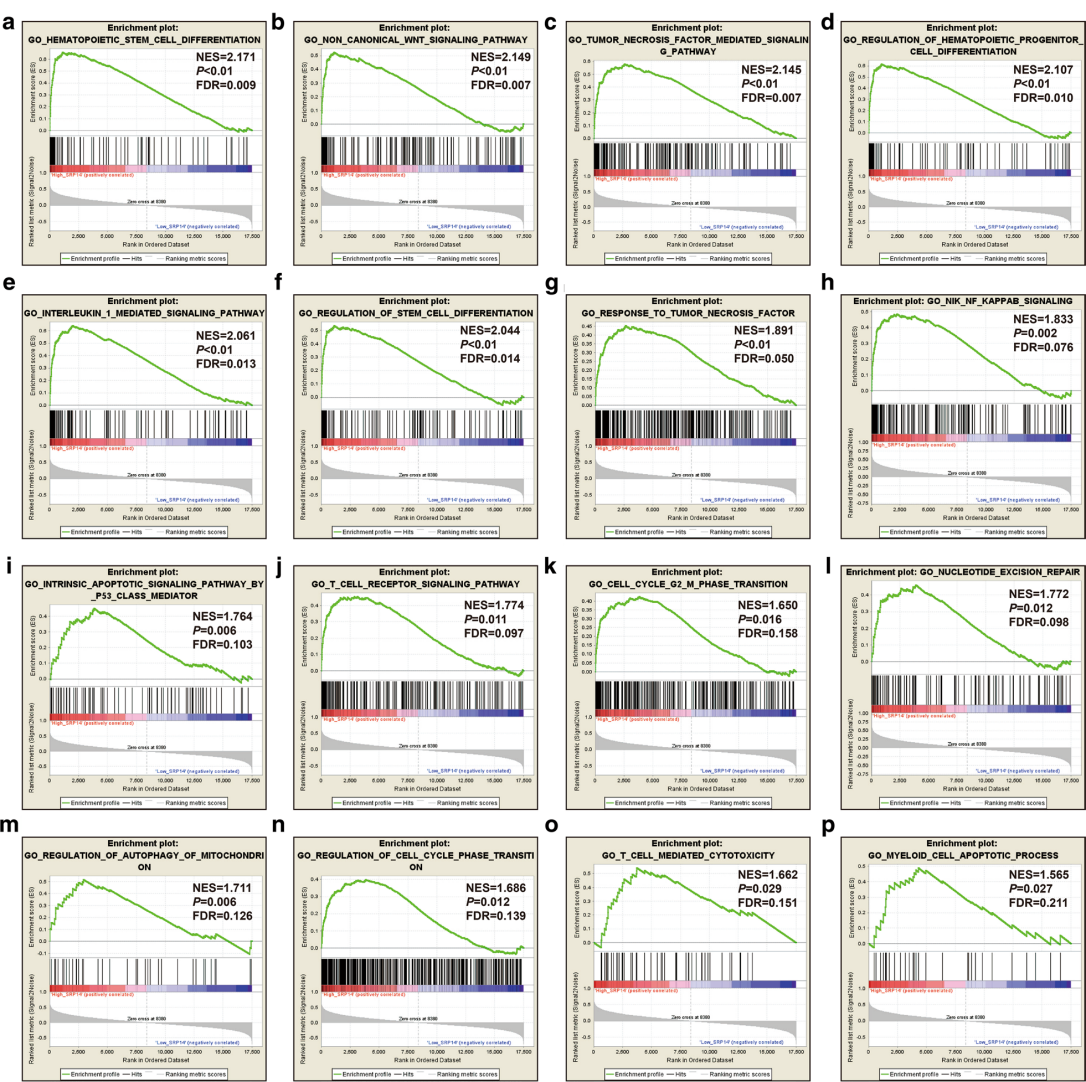
GSEA analysis between low and high SRP14 phenotypes in the TCGA cohort using the C5 reference gene set. **a** Hematopoietic stem cell differentiation; **b** non-canonical Wnt signaling pathway; **c** TNF-mediated signaling pathway; **d** regulation of hematopoietic progenitor cell differentiation; **e** IL-1-mediated signaling pathway; **f** regulation of stem cell differentiation; **g** response to TNF; **h** NIK/NF-κB signaling; **i** intrinsic apoptotic signaling pathway by p53 class mediator; **j** TCR signaling pathway; **k** cell cycle G2/M phase transition; **l** nucleotide excision repair; **m** regulation of autophagy of mitochondria; **n** regulation of cell cycle phase transition; **o** T cell-mediated cytotoxicity and myeloid cell apoptotic process; and **p** myeloid cell apoptotic process

**Fig. 9:**
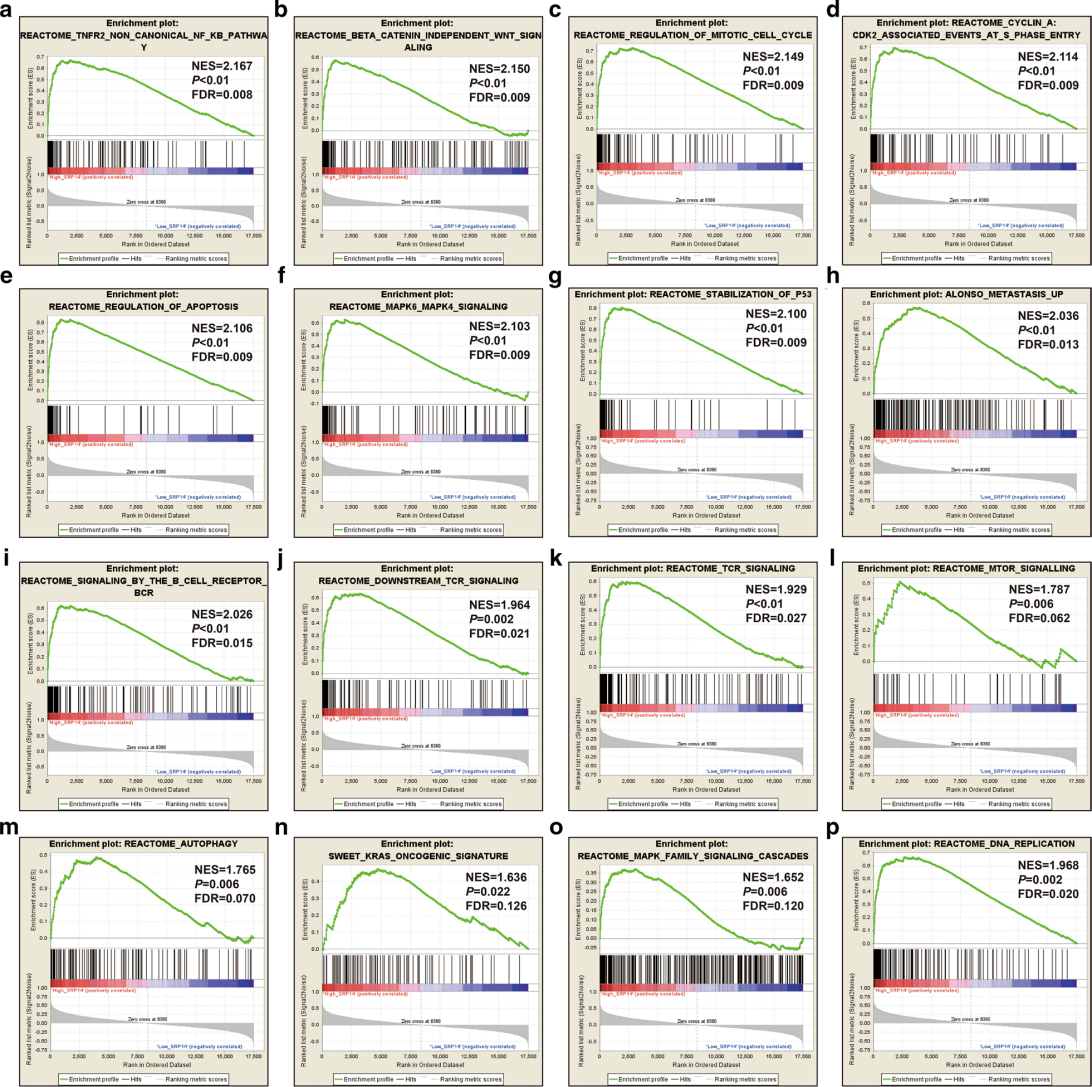
GSEA analysis between low and high SRP14 phenotypes in TCGA cohort using the C2 reference gene set. **a** TNFR2 noncanonical NF-kB pathway; **b** β-catenin-independent Wnt signaling; **c** regulation of mitotic cell cycle; **d** cyclin A/cyclin-dependent kinase 2-associated events at S phase entry; **e** regulation of apoptosis; **f** MAPK6/MAPK4 signaling; **g** stabilization of p53; **h** metastasis-up; **i** B cell receptor signaling; **j** downstream TCR signaling; **k** TCR signaling; **l** mTOR signaling; **m** autophagy; **n** KRAS oncogenic signature; **o** MAPK family signaling cascades; and **p** DNA replication

We further explored the functional mechanisms of SRP14 in AML using GSEA. Using the C5 reference gene set in TCGA, we found that high SRP14 expression phenotype was significantly involved in pathways including hematopoietic stem cell differentiation, noncanonical Wnt signaling, TNF-mediated signaling, regulation of hematopoietic progenitor cell differentiation, IL-1-mediated signaling, regulation of stem cell differentiation, response to TNF, NIK/NF-κB signaling, intrinsic apoptotic signaling by p53 class mediator, TCR signaling, cell cycle G2/M phase transition, nucleotide excision repair, regulation of autophagy of mitochondria, regulation of cell cycle phase transition, T cell mediated cytotoxicity, and myeloid cell apoptotic (Fig. 8a–p, Additional file 2: Table S6). Using the C2 reference gene set, we found significant differences in the following signaling pathways between the high- and low-SRP14 expressing phenotypes: TNF receptor-2 non-canonical NF-kB pathway, β-catenin-independent Wnt signaling, regulation of mitotic cell cycle, cyclin A/cyclin-dependent kinase 2-associated events at S phase entry, regulation of apoptosis, MAPK6/MAPK4 signaling, stabilization of p53, metastasis-up, signaling by the B cell receptor signaling (BCR), downstream TCR signaling, TCR signaling, mTOR signaling, autophagy, KRAS oncogenic signature, MAPK family signaling cascades and DNA replication (Fig. 9a–p, Additional file 2: Table S7).

**Fig. 10:**
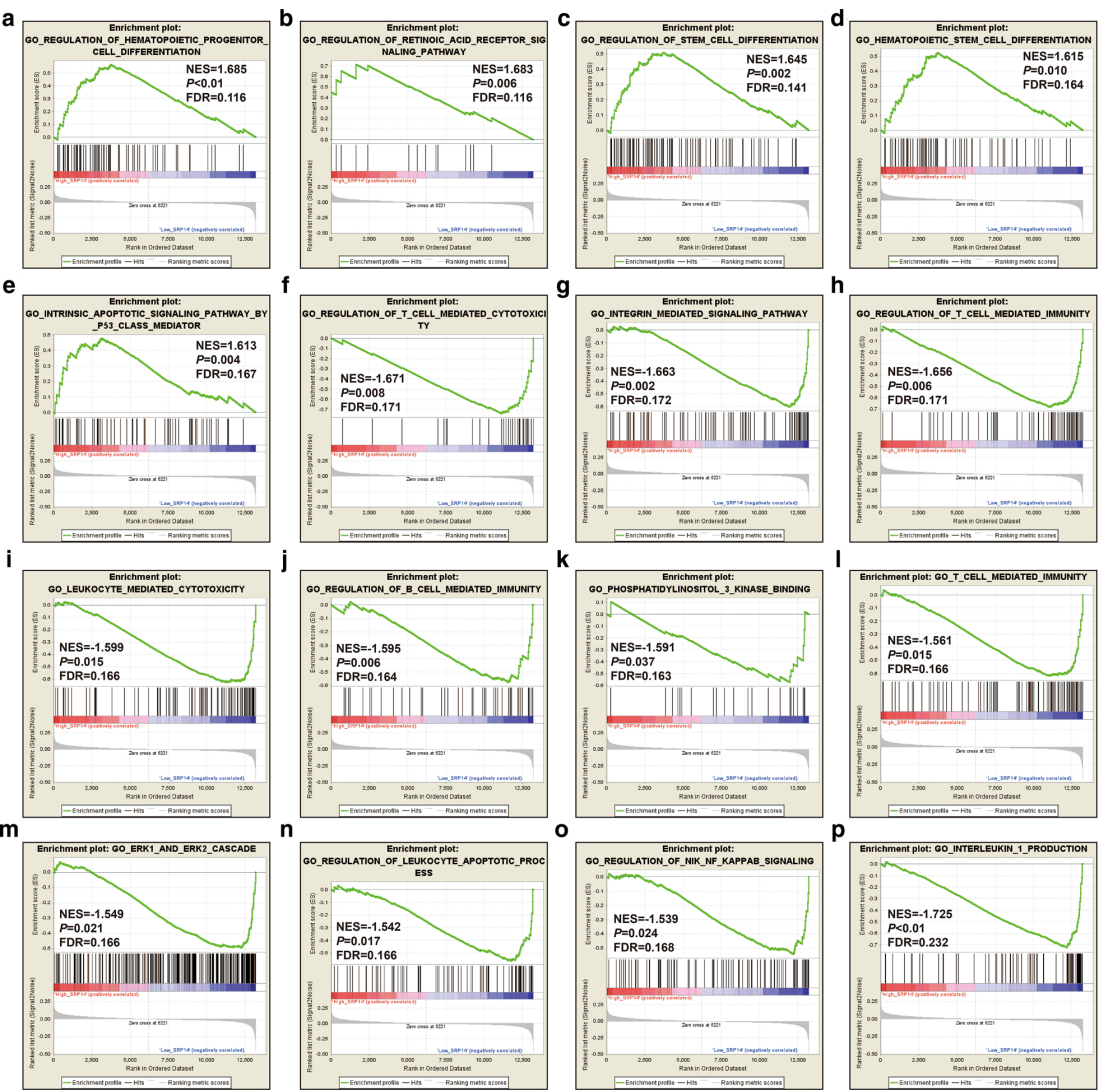
GSEA analysis between low and high SRP14 phenotypes in GSE12417 cohort using the C5 reference gene set. **a** Regulation of hematopoietic progenitor cell differentiation; **b** regulation of acid receptor signaling pathway; **c** regulation of stem cell differentiation; **d** hematopoietic stem cell differentiation; **e** intrinsic apoptotic signaling pathway by p53 class mediator; **f** regulation of T cell mediated cytotoxicity; **g** integrin-mediated signaling pathway; **h** regulation of T cell-mediated immunity; **i** leukocyte-mediated cytotoxicity; **j** regulation of B cell-mediated immunity; **k** phosphatidylinositol 3 kinase binding; **l** T cell-mediated immunity; **m** Erk1 and Erk2 cascade; **n** regulation of leukocyte apoptotic process; **o** regulation of NIK/NF-κB signaling; and **p** IL-1 production

**Fig. 11:**
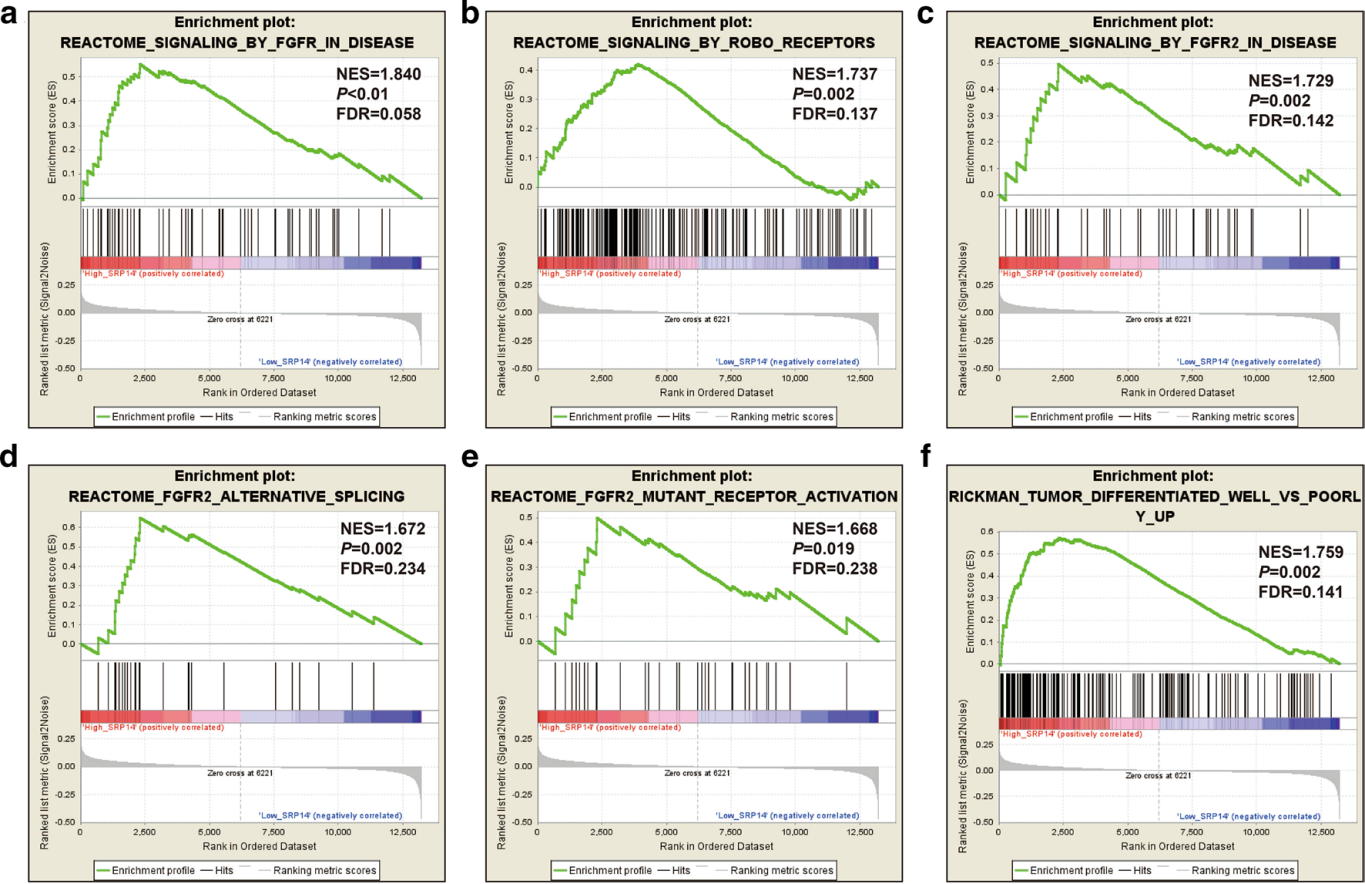
GSEA analysis between low and high SRP14 phenotypes in GSE12417 cohort using the C2 reference gene set. **a** FGFR in disease; **b** signaling by ROBO receptors; **c** signaling by FGFR2 in disease; **d** FGFR2 alternative splicing; **e** FGFR2 mutant receptor activation; and **f** Tumor differentiated well vs poorly up

We verified the above findings using the GSE12417 dataset as a validation cohort. Using the C5 reference gene set, regulation of the following biological processes differed significantly between the high and low SRP14 expression phenotypes: hematopoietic progenitor cell differentiation, regulation of acid receptor signaling pathway, regulation of stem cell differentiation, hematopoietic stem cell differentiation, intrinsic apoptotic signaling pathway by p53 class mediator, regulation of T cell mediated cytotoxicity, integrin-mediated signaling, regulation of T cell-mediated immunity, leukocyte-mediated cytotoxicity, regulation of B cell-mediated immunity, phosphatidylinositol 3 kinase (PI3K) binding, T cell-mediated immunity, Erk1 and Erk2 cascade, regulation of leukocyte apoptotic process, regulation of NIK/NF-κB signaling, and IL-1 production (Fig. 10a–p, Additional file 2: Table S8). GSEA results using the C5 reference gene set identified signaling by FGFR in disease, signaling by Robo receptors, signaling by FGFR2 in disease, FGFR2 alternative splicing, FGFR2 mutant receptor activation, and tumor differentiated well vs poorly up (Fig. 11A-F, Additional file 2: Table S9).

### Targeted drug screening of SRP14 in AML

**Fig. 12:**
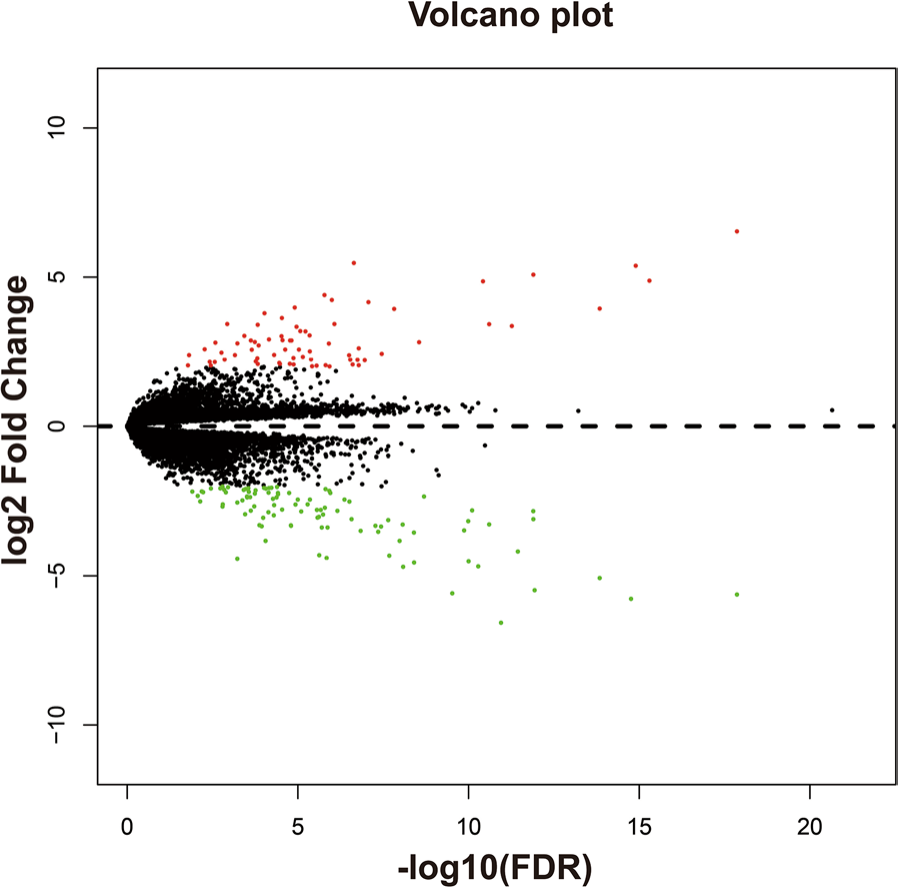
Volcano plot of DEGs between low and high SRP14 phenotypes in TCGA cohort

**Fig. 13:**
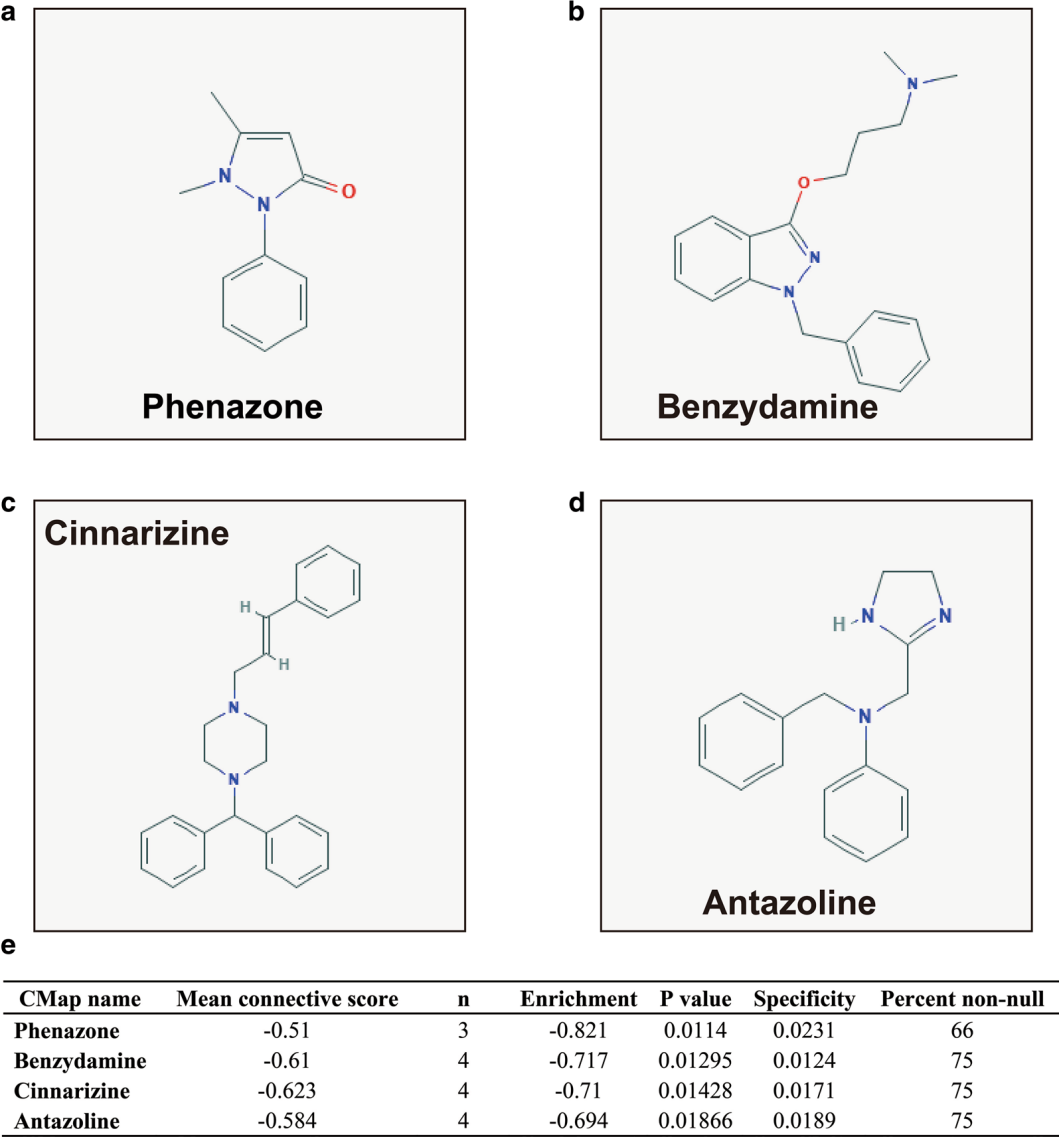
CMap analysis results for low and high SRP14 phenotypes in TCGA cohort. **a** phenazone; **b** benzydamine; **c** cinnarizine; **d** antazoline; and **e** CMap analysis results

**Fig. 14:**
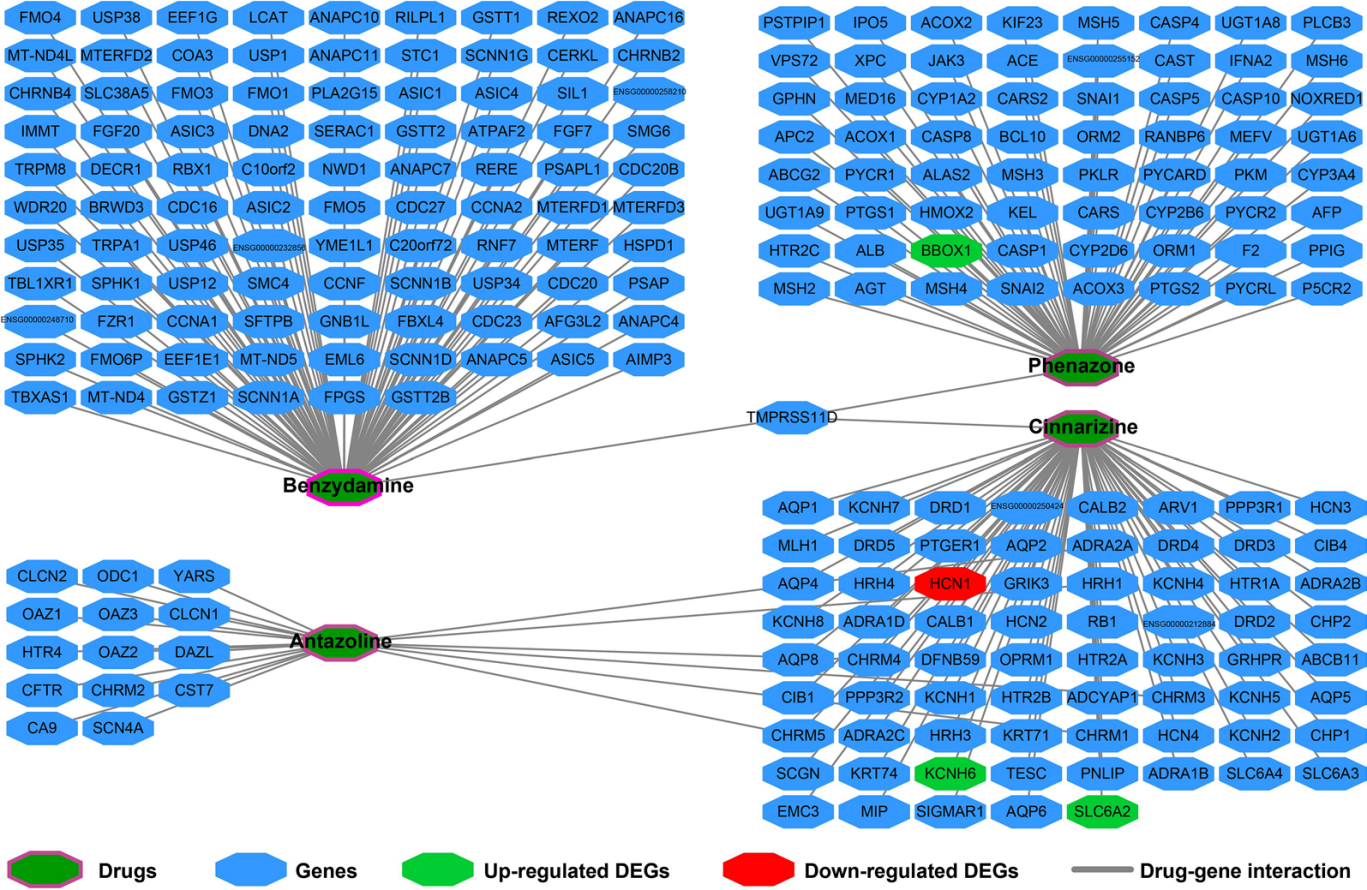
Drug–gene interaction networks generated from STITCH

Using the AML cohort in the TCGA genome-wide dataset, we identified170 DEGs between the high and low SRP14 expression groups (Additional file 2: Table S10, Fig. 12, Additional file 1: Figure S1). CMap was then used for targeted drug screening, and four drugs were identified as potential SRP14-targeted drugs for AML. The chemical structures and CMap results for the four drugs are summarized in Fig. 13a–e. The subsequent drug–gene interaction networks showed that cinnarizine could participate in the targeted therapy of SRP14 in AML by regulating hyperpolarization-activated cyclic nucleotide gated potassium channel 1 (HCN1), potassium voltage-gated channel subfamily H member 6 (KCNH6), and solute carrier family 6 member 2 (SLC6A2), all of which genes were differentially expressed between the two SRP14 expression phenotypes. (Fig. 14). Phenazone could act by regulating the DEG for gamma-butyrobetaine hydroxylase 1 (BBOX1). We aimed to verify these results found in TCGA cohort using the GSE12417 validation cohort. Unfortunately, however, no DEGs meeting our criteria were detected in the GSE12417 cohort (Additional file 2: Table S11).

## Discussion

A search of the literature found no previous studies reporting the role of SRP14 expression in the prognosis of AML. We therefore explored the prognostic value of SRP14 in AML using two independent cohorts, and screened for and identified the biological functional mechanisms of SRP14 in AML by bioinformatics analysis of a genome-wide expression profile dataset. Using genome-wide co-expression gene screening, we determined a large number of biological processes and signaling pathways that were enriched through SRP14 and its co-expression genes, and which might be involved in the mechanisms of SRP14 functions in AML. Cell cycle and adhesion are the most common biological processes in tumor cells. Cell adhesion has been closely related to tumor metastasis and drug resistance, while the cell cycle is closely related to malignant phenotypes of tumor proliferation [14–16]. Cellular adhesion molecules also affect the prognosis of AML and can be used as potential targets for AML-targeted therapy [17]. Abou et al. also concluded that cell cycle inhibitors could be used as a complementary treatment for AML-targeted therapy and chemotherapy, to help improve the treatment outcomes of AML patients [18]. Regarding the MAPK signaling pathway in AML, mesenchymal stromal cells can regulate the apoptosis repressor with caspase recruitment domain in AML by activating MAPK and PI3K signaling pathways, thereby affecting drug resistance and prognosis, and can thus be used as a potential therapeutic target for AML [19]. Dong et al. suggested that TNF-α expression was significantly increased in AML patients, and ROC curve analysis indicated that it could be used to differentiate between AML and nonleukemia samples [20]. TCR is widely used in the treatment of hematologic malignancies. T cells can be designed to express tumor-associated antigens derived from intracellular or cell surface proteins for the treatment of hematologic malignancies [21]. TCR gene therapy targeting Wilms’ tumor antigen 1 prevented the recurrence of AML after transplantation [22]. Mahmud et al. investigated the molecular mechanisms of childhood AML by peptide microarray and found that DNA damage response and repair were involved in its recurrence [23]. DNA damage and changes in DNA damage response are important characteristics of genetic instability closely related to the pathogenesis of AML. In addition, DNA damage response kinase can be a potential therapeutic target for AML [24, 25]. NF-κB pathways, as an important tumor-related mechanism, play a key role in many cellular functions, including cancer cell apoptosis, proliferation, angiogenesis, and immune-related functions [26], and can also be used as a therapeutic target for AML [26, 27].

NF-κB and TCR were also significantly enriched according to the GSEA enrichment results. The p53 gene is critical for hematopoietic stem cell function and its dysfunction can affect the evolution, biological phenotype, treatment response, and prognosis of AML [28–31]. Evaluation of the function of p53 protein is conducive to accurate p53-based targeted therapy for AML [28]. Hayashi et al. found that drug-induced activation of the p53 gene in AML and the combination of immunosuppressive programmed death-ligand 1 could have a significant anti-AML effect [32]. p53 gene mutations in AML patients often predict a poor prognosis, and are associated with a low chemotherapy-response rate and long-term survival after allogeneic hematopoietic stem cell transplantation [33–35]. Folkerts et al. developed a treatment strategy for AML patients with wild-type p53 by targeting autophagy- related genes [36]. Carey et al. found that IL-1 promoted the growth of myeloid progenitor cells in AML while inhibiting the proliferation of normal progenitor cells [37]. Hematopoietic progenitor cell differentiation abnormalities can lead to the occurrence of AML. In this study, we found differences in genes related to hematopoietic progenitor cell differentiation between AML patients with different SRP14 expression phenotypes, suggesting that SRP14 may be related to the pathogenesis of AML. We also identified enrichment of numerous signaling pathways and biological processes closely related to cancers through GSEA in both cohorts. However, these results need further verification.

Previous studies suggested that phenazone could be used as a marker of liver metabolic function and to evaluate the effects of chemotherapy in AML patients [38], and as a biomarker of liver metabolic efficiency in breast cancer [39]. Benzydamine has previously been reported to have a therapeutic role in patients with oral mucositis undergoing radiotherapy [40–44], while cinnarizine has been widely reported to be closely related to tumors. Fagone et al. identified cinnarizine as a potential therapeutic drug for metastatic uveal melanoma using drug-prediction tools based on whole-genome datasets [45], while Astin et al. screened cinnarizine in zebrafish models as a potential new inhibitor for cancer lymphangiogenesis and lymphatic-mediated cancer metastasis [46]. Cinnarizine also affected the function of radiation and could be used as a radiosensitizer for murine tumors [47, 48]. Allen et al. found that cinnarizine inhibited mammalian target of rapamycin (mTOR) protein and mTOR complex 1 for anti-tumor therapy [49]. Deka et al. screened and identified cinnarizine as an anti-cancer drug for breast cancer using an integrating virtual screening biochemical experimental approach [50]. In the treatment of hematologic diseases, Schmeel et al. showed that cinnarizine selectively induced apoptosis of myeloma and lymphoma cells by inhibiting Wnt signaling, but had no effect on normal cells [51]. However, we were unable to find any studies reporting on the function of antazoline in cancers.

This study had some limitations. First, we failed to screen eligible DEGs in the GSE12417 cohort for subsequent CMap analysis, and the screened drugs thus need to be verified in future studies. Second, the sample size was limited, and the prognostic value of SRP14 in AML needs to be verified in future large, multicenter studies. Third, the potential biological functions and mechanisms of SRP14 in AML identified in this study remain to be further validated by in vitro and in vivo experiments.

## Conclusions

The results of the present study showed that high SRP14 expression was significantly related to a poor prognosis and may serve as a prognostic biomarker in patients with AML. Genome-wide co-expression analysis suggested that SRP14 might play a role in AML by participating in the regulation of biological processes and signaling pathways such as cell cycle, cell adhesion, MAPK, TNF, TCR, DNA damage response, and NF-κB signaling. GSEA indicated that SRP14 was significantly enriched in biological processes and signaling pathways, such as regulation of hematopoietic progenitor cell differentiation and stem cell differentiation, intrinsic apoptotic signaling by p53 class mediator, IL-1, T cell-mediated cytotoxicity, and NIK/NF-κB signaling. Using the TCGA AML dataset, we also identified phenazone, benzydamine, cinnarizine, and antazoline as potential SRP-targeted therapeutic drugs in AML. However, the results of this study need to be verified by further in vitro and in vivo experiments.

## Supplementary Information

**Additional file 1: Figure S1.** Heat map of DEGs between low and high SRP14 phenotypes in TCGA cohort

**Additional file 2: Table S1.** Co-expressed genes of SRP14 in TCGA AML cohort. **Table S2.** Co-expressed genes of SRP14 in GSE12417 AML cohort. **Table S3.** Functional enrichment results of SRP14 and its co-expressed genes in TCGA AML cohort. **Table S4** Functional enrichment results of SRP14 and its co-expressed genes in GSE12417 AML cohort. **Table S5**. Functional enrichment analysis of 44 positive correlation genes common to both cohorts. **Table S6**. GSEA analysis between low and high SRP14 phenotypes in TCGA cohort using the C5 reference gene set. **Table S7**. GSEA analysis between low and high SRP14 phenotypes in TCGA cohort using the C2 reference gene set. **Table S8**. GSEA analysis between low and high SRP14 phenotypes in GSE12417 cohort using the C5 reference gene set. **Table S9**. GSEA analysis between low and high SRP14 phenotypes in GSE12417 cohort using the C2 reference gene set. **Table S10**. DEGs for TCGA cohort. **Table S11**. DEGs for GSE12417 cohort

## Data Availability

The raw datasets used during the present study can be downloaded from The Cancer Genome Atlas (https://portal.gdc.cancer.gov/projects/TCGA-LAML) and GSE12417 (https://www.ncbi.nlm.nih.gov/geo/query/acc.cgi?acc=GSE12417). Both the AML RNA-seq and GSE12417 expression profile microarray datasets are open access and can be downloaded directly from the corresponding database without any login account.

## Abbreviations

SRP14: Signal recognition particle 14
AML: Acute myeloid leukemia
TCGA: The Cancer Genome Atlas
NF-κB: Nuclear factor-kappa B
GEO: Gene expression omnibus
GSEA: Gene set enrichment analysis
GO: Gene Ontology
FDR: False discovery rate
FC: Fold change
DEG: Differentially expressed gene
CMap: Connectivity Map
HR: Hazard ratio
CI: Confidence interval
OS: Overall survival
MST: Median survival time
ROC: Receiver operating characteristic
AUC: Area under the curve
