# Supplementary material for: Identification and validation of signal recognition particle 14 as a prognostic biomarker predicting overall survival in patients with acute myeloid leukemia
Source: BMC Med Genomics. 2021 May 13;14:127. doi: 10.1186/s12920-021-00975-2 (PMC8120815; doi:10.1186/s12920-021-00975-2)
Supplement: Supplementary file 1 — Additional file 1: Figure S1. Heat map of DEGs between low and high SRP14 phenotypes in TCGA cohort [file 12920_2021_975_MOESM1_ESM.pdf]

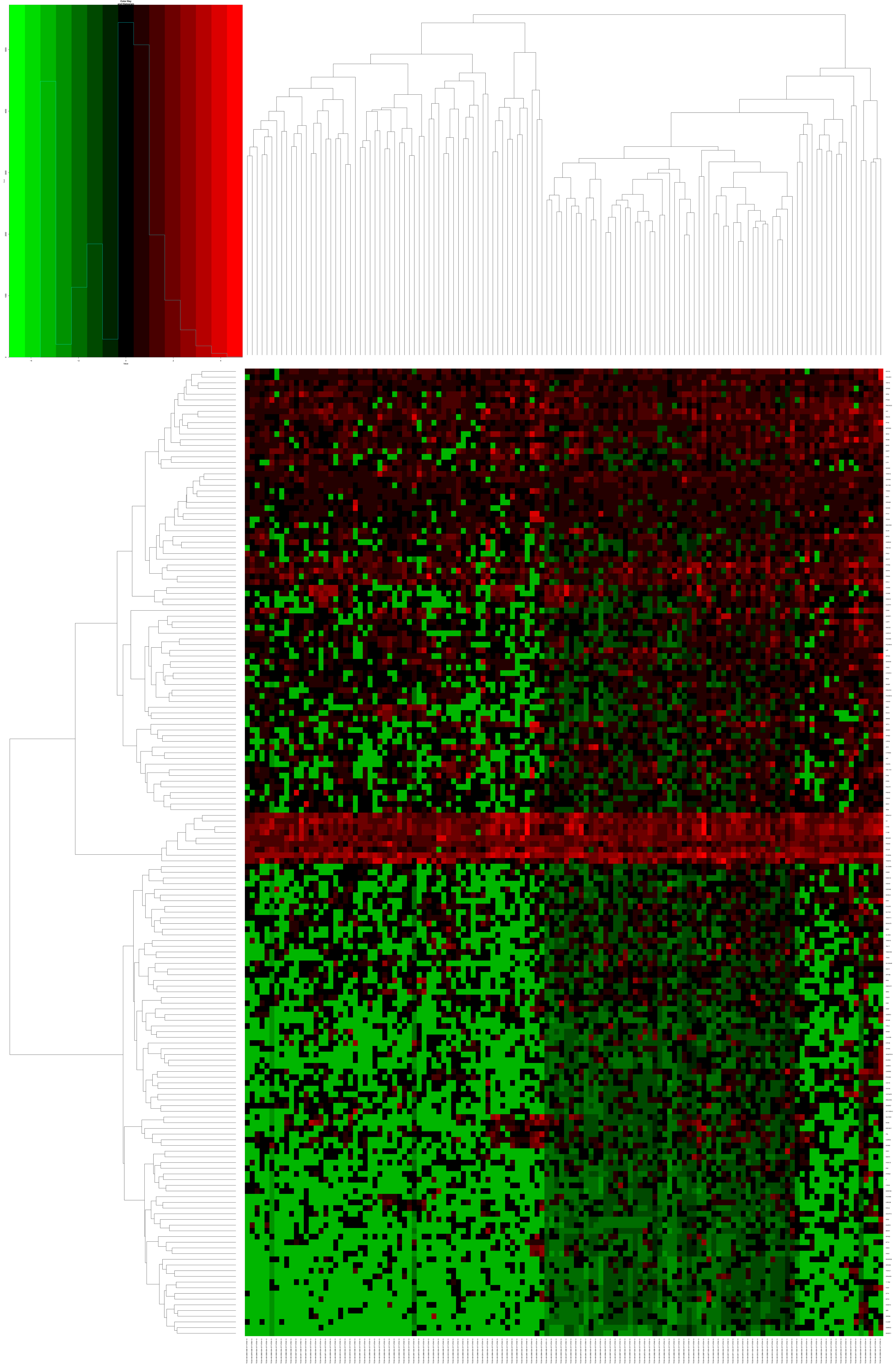

Figure S1. Heat map of DEGs of TCGA cohort.
